# Phenotypes of Hypertension: Impact of Age and Sex on Hemodynamic Mechanisms

**DOI:** 10.1161/JAHA.125.042096

**Published:** 2025-08-29

**Authors:** Domonkos Cseh, Yasmin, Barry J. McDonnell, Kaisa M. Mäki‐Petäjä, Taylor Simonian, John R. Cockcroft, Ian B. Wilkinson, Carmel M. McEniery, ACCT Investigators, John R. Cockcroft, Zahid Dhakam, Stacey S. Hickson, Julia Howard, Kaisa M. Mäki‐Petäjä, Barry J. McDonnell, Carmel M. McEniery, Karen Miles, Maggie Munnery, Pawan Pusalkar, Christopher Retallick, Jane Smith, Edna Thomas, Sharon Wallace, Ian B. Wilkinson, Susannah Williams, Jean Woodcock‐Smith, Yasmin

**Affiliations:** ^1^ Division of Experimental Medicine and Immunotherapeutics, Department of Medicine University of Cambridge Cambridge UK; ^2^ Centre for Cardiovascular Research, Innovation and Development Cardiff Metropolitan University Cardiff UK

**Keywords:** arterial stiffness, cardiac output, hypertension, hypertensive phenotypes, peripheral vascular resistance, High Blood Pressure, Hypertension, Hemodynamics, Women, Aging

## Abstract

**Background:**

Essential hypertension is often treated as a uniform condition. However, it encompasses distinct patterns of blood pressure elevation such as isolated systolic, systolic diastolic, and isolated diastolic hypertension, which vary in prevalence according to age and sex. We hypothesized that different hemodynamic mechanisms account for the age‐ and sex‐related differences in these hypertensive phenotypes.

**Methods:**

In this cross‐sectional analysis of the ACCT (Anglo‐Cardiff Collaborative Trial), 5371 individuals (2402 male), aged 18 to 92 years, free of cardiovascular disease and medication were included. Blood pressure, cardiac output, stroke volume, peripheral vascular resistance (PVR), and aortic pulse wave velocity were measured. Within each sex, subjects were stratified according to age (<30, 30–60, and >60 years), and blood pressure phenotype based on clinic (seated) blood pressure.

**Results:**

Isolated systolic hypertension was the most common hypertensive phenotype in young men and characterized by an elevated cardiac output and stroke volume. In contrast, systolic diastolic hypertension and isolated diastolic hypertension were more common in younger females, with systolic diastolic hypertension associated with elevated PVR and aortic pulse wave velocity, and isolated diastolic hypertension with increased PVR. Systolic diastolic hypertension was the most common phenotype in middle age and accompanied by increased PVR in both sexes. Isolated systolic hypertension was again the most common phenotype in older individuals. However, in contrast to younger adults, isolated systolic hypertension affected both men and women equally (~1:1) and was characterized by elevated aortic pulse wave velocity and PVR.

**Conclusions:**

Different hypertensive phenotypes are characterized by distinct hemodynamic mechanisms in an age‐ and sex‐dependent manner. Targeting therapy toward primary hemodynamic abnormalities could lead to more effective interventions in essential hypertension.

**Nonstandard Abbreviations and Acronyms**
AIx: augmentation index
aPWV: aortic pulse wave velocity
CI: cardiac index
CO: cardiac output
IDH: isolated diastolic hypertension
ISH: isolated systolic hypertension
PVRI: peripheral vascular resistance index
SDH: systolic‐diastolic hypertension
SV: stroke volume

**Clinical Perspective**

**What Is New?**

- Our study is the first to examine the major hemodynamic mechanisms behind different hypertensive phenotypes across different age groups and sexes using consistent methods in a sufficiently large population.
- We demonstrated that the hemodynamic mechanisms underlying different hypertensive phenotypes differ significantly between sexes—particularly in young adults—and vary across the adult lifespan.

**What Are the Clinical Implications?**

- Our results could represent an important step in moving away from the concept of treating essential hypertension as a uniform condition and toward tailoring therapy to the age‐, sex‐, and phenotype‐specific hemodynamic abnormalities, which could play a key role in improving blood pressure control.

Essential hypertension is a common condition and the leading risk factor for death globally.^1^ Despite this, only a minority of patients are appropriately identified and treated to reach recommended blood pressure (BP) targets.^2^, ^3^, ^4^, ^5^, ^6^, ^7^ Although often treated as a uniform condition, essential hypertension encompasses a number of distinct patterns of BP elevation such as isolated systolic (ISH), systolic diastolic (SDH), and isolated diastolic hypertension (IDH), which differ in prevalence, depending on age. Indeed, data from the Framingham Heart Study in >2700 individuals show that the prevalence of ISH is lowest in adults in their 30s but increases thereafter.^8^ In contrast, the proportion of patients with IDH declines with age whereas SDH is predominant in middle age.^9^ More recent studies have shown that ISH is also the most common form of hypertension in adolescents and young adults.^10^, ^11^, ^12^, ^13^

Previous investigations have demonstrated that elevated cardiac output (CO) and stroke volume (SV) are dominant hemodynamic mechanisms underlying ISH in young adults,^11^ whereas increased arterial stiffness is associated with ISH in older individuals.^14^, ^15^ Earlier investigations in essential hypertension, involving mostly middle‐aged individuals, describe a predominantly SDH phenotype characterized by normal or low CO or cardiac index (CI), but increased peripheral vascular resistance (PVR).^16^, ^17^ Taken together, these studies suggest that the factors underlying the different hypertension phenotypes also vary according to age. However, a detailed analysis of the hemodynamic variables associated with each hypertensive phenotype and whether these differ by sex, has not been undertaken within a single, large population, covering the adult age span, using consistent methodologies throughout. We hypothesized that different hemodynamic mechanisms account for the observed age‐ and sex‐related differences in hypertensive phenotypes and our aim was to test this hypothesis in a large population of healthy individuals, covering the adult age span.

## METHODS

The data that support the findings of this study are available from the corresponding author upon reasonable request.

### Participants

Subjects were drawn from the ACCT (Anglo‐Cardiff Collaborative Trial) population, which consists of >12 000 individuals, selected at random from local general practice lists and open‐access Cardiovascular Risk Assessment Clinics across East Anglia and Wales. The study population has been described in detail elsewhere.^18^ For the current analyses, subjects with secondary hypertension, diabetes, a serum cholesterol ≥6.5 mmol/L, renal disease (defined as a clinical history, creatinine ≥150 μmol/L, or an active urinary sediment), or cardiovascular disease (defined as a clinical history or evidence on examination) were excluded from the analysis, as were subjects receiving any vasoactive medication. These exclusion criteria provided the opportunity to focus on the primary hemodynamic abnormalities underlying the different hypertensive phenotypes, without the confounding influence of the aforementioned pathological conditions. This yielded a total of 5371 individuals in whom detailed hemodynamic data were available. Approval for all studies was obtained from the Local Research Ethics Committees, and written informed consent obtained from each participant.

### Protocol

Measurements were performed in a quiet, temperature‐controlled room (22–24 °C). Height and weight were assessed. After at least 5 minutes of seated rest, brachial (clinic) BP was measured. Following a further 10 minutes of supine rest, BP was remeasured and radial, carotid, and femoral artery waveforms recorded. CO and SV were then assessed and PVR calculated, as described subsequently.

### Hemodynamic Measurements

Brachial systolic and diastolic BP (SBP and DBP, respectively) were recorded in the dominant arm using a validated oscillometric technique (HEM‐705CP; Omron Corporation, Kyoto, Japan), with the average of the final 2 measurements used for analysis. Radial artery waveforms were recorded with a high fidelity micromanometer (SPC‐301; Millar Instruments, Texas) from the wrist of the dominant arm. The radial artery waveform was then used to generate a corresponding central (ascending aortic) waveform through use of a validated generalized transfer function (SphygmoCor; AtCor Medical, Sydney, Australia).^19^ Pulse wave analysis of the central waveform was used to measure aortic augmentation index (AIx) and heart rate, whilst mean arterial pressure (MAP) was obtained through integration of the waveform, using the system software. The aortic pulse wave velocity (aPWV) was measured using the same device by sequentially recording ECG‐gated carotid and femoral artery waveforms, as described previously.^20^ The distance for the determination of aPWV was measured using a tape measure as the distance between the suprasternal notch and the femoral site minus the distance between the suprasternal notch and the carotid site. CO and SV were assessed using a noninvasive, inert gas rebreathing technique.^21^, ^22^ Briefly, while resting, subjects continuously rebreathed a gas mixture (1% SF_6_, 5% N_2_0, and 94% O_2_) over 20 seconds, with a breathing rate of 15 breaths/min. Expired gases were sampled continuously and analyzed by an infrared photoacoustic gas analyzer (Innocor; Innovision A/S, Glamsbjerg, Denmark). PVR was calculated from the formula:

$$\mathrm{PVR}\;\left(\mathrm{dyn}\;s\;{\mathrm{cm}}^{-5}\right)=\mathrm{MAP}\;\left(\mathrm{mm}\;\mathrm{Hg}\right)\times 80/\mathrm{CO}\;\left(L/\min\right).$$

Inert gas rebreathing was performed once. Other measurements were made in duplicate, and the mean values were used in the analyses. All measurements were performed by trained investigators.

### Statistical Analysis

Data were analyzed using SPSS software v. 29 (IBM Corporation, Somers, NY). Subjects were grouped into 4 categories, according to seated brachial BP: nonhypertensive (SBP <140 mm Hg and DBP <90 mm Hg), ISH (SBP ≥140 mm Hg and DBP <90 mm Hg), SDH (SBP ≥140 mm Hg and DBP ≥90 mm Hg), and IDH (SBP <140 mm Hg and DBP ≥90 mm Hg). In order to provide an overview of the relationship between the hypertensive phenotypes and arterial hemodynamics, data were grouped into 3 age groups (<30, 30–60, and >60 years), and analyzed separately for men and women. Differences in the distribution of hypertensive phenotypes across age ranges between men and the overall population, and between women and the overall population were examined using chi‐squared goodness‐of‐fit test. Between‐group comparisons were performed using the following tests: (1) One‐way ANOVA with Bonferroni post hoc test for variables with normal distributions and equal variances across the examined groups; (2) Welch's ANOVA with Games–Howell post hoc test for variables with normal distributions but unequal variances across the examined groups; and (3) Kruskal–Wallis test with Dunn–Bonferroni post hoc test for variables with skewed distributions. In subsequent analyses, CO, SV, and PVR were adjusted for body surface area (providing the following variables: CI, stroke volume index and peripheral vascular resistance index [PVRI], respectively), aPWV for MAP and AIx for height and heart rate. There were no missing data for the stratifying variables (age, sex, clinic BP). As an additional exploratory analysis, we further subdivided our group without hypertension into participants with optimal BP (SBP <120 mm Hg and DBP <80 mm Hg) and participants with prehypertension (the remaining individuals without hypertension). We then repeated our analyses using 5 groups—optimal BP, prehypertension, ISH, SDH, IDH—to assess the robustness of our findings and to explore hemodynamic patterns in the population with prehypertension. The proportion of missing data was ≤3% for all other variables. Missing data for these variables were treated as missing at random and excluded on an analysis‐by‐analysis basis. Data are presented as means±SD, and *P*<0.05 was considered significant.

## RESULTS

**Figure 1:**
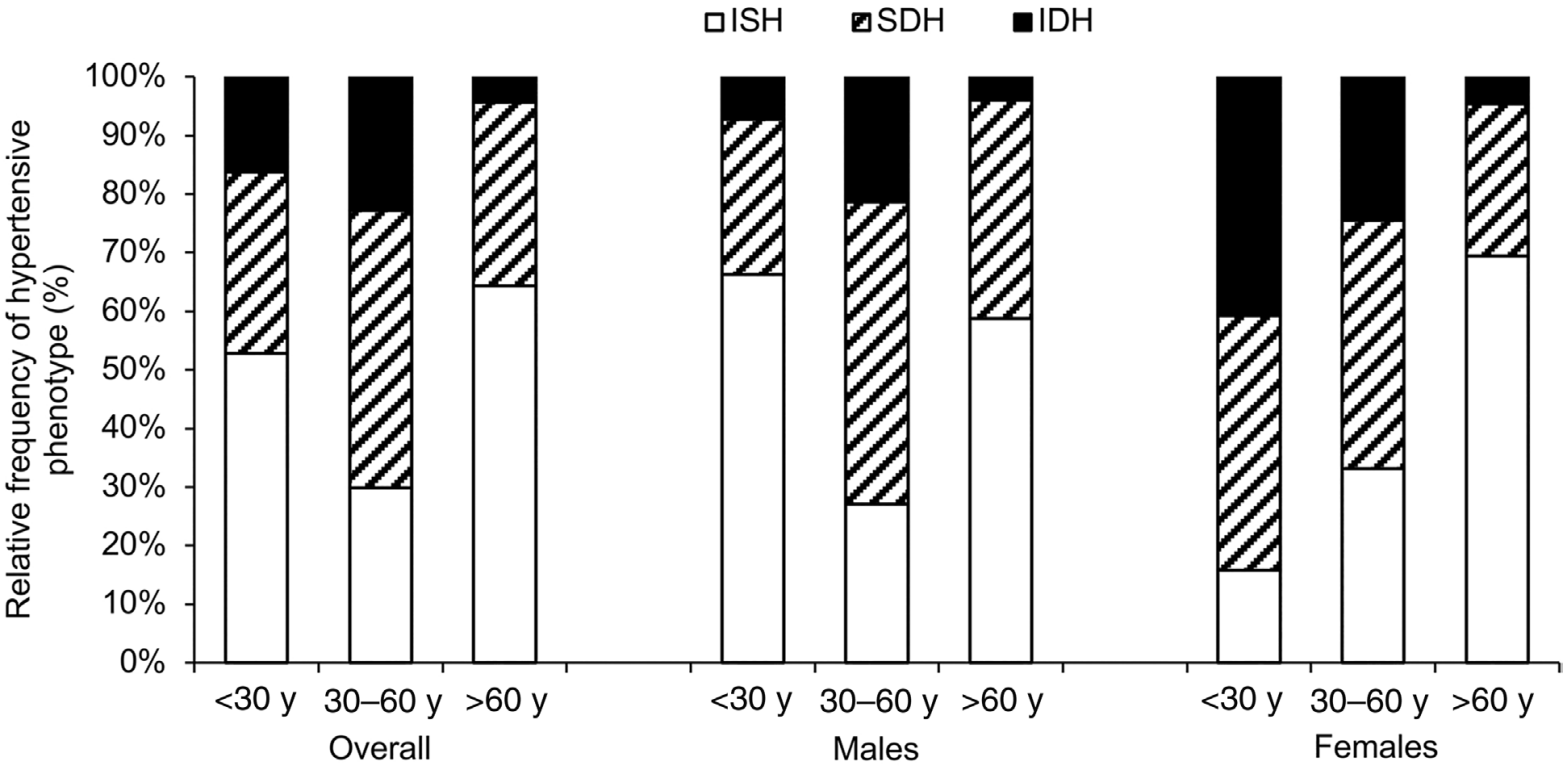
Frequency distribution of hypertensive individuals according to blood pressure phenotype, stratified by age and sex. IDH indicates isolated diastolic hypertension; ISH, isolated systolic hypertension; and SDH, systolic diastolic hypertension.

Figure 1 shows the distribution of individuals according to hypertensive phenotype, for each age group and sex, on the basis of seated BP. The overall prevalence of hypertension, irrespective of its form, within the population was 27.6% (ISH=14.5%, n=780; SDH=9.9%, n=530; and IDH=3.2%, n=173). Further information about the distribution of individuals according to BP categories is presented in Table S1A. Table S1B shows that the distribution of different hypertensive phenotypes across age ranges in men is different compared with the overall population, and the same is true for women. In the <30s, ISH was most common in men, whereas in women, SDH and IDH were more common. In both men and women, SDH was the most predominant form of hypertension between 30 and 60 years, with ISH the most common form in the >60s, with no preponderance of men over women, unlike in young adults. Table 1 shows the anthropometric and seated BP data of each group. In the <30s, both male and female individuals with SDH were older compared with individuals without hypertension; men with any phenotype of hypertension and women with SDH and IDH had increased body mass index (BMI) compared with nonhypertensive participants. In the middle age group, female participants with ISH were older than women in other groups; male participants with SDH and IDH and women with any phenotype of hypertension had increased weight and BMI compared with individuals without hypertension. In the >60s, both male and female participants with ISH were older compared with the other groups; men with any phenotype of hypertension and women with ISH had increased BMI compared with women without hypertension.

**Table 1:** Anthropometric and Seated Blood Pressure Data According to Blood Pressure Phenotype in Men and Women

		Nonhypertensive	ISH	SDH	IDH	*P* value
**<30 y**	**Male**	**n=874**	**n=138**	**n=55**	**n=15**	
	**Female**	**n=1056**	**n=12**	**n=33**	**n=31**	
Age, y	Male	21±3	21±3	23±4*, ||	23±4	<0.001¶
	Female	21±3	21±3	24±4*	22±3	<0.001¶
Height, m	Male	1.79±0.07	1.81±0.07*	1.77±0.08||	1.80±0.05	0.003*
	Female	1.66±0.07	1.68±0.09	1.63±0.06	1.62±0.05*	0.003*
Weight, kg	Male	75±12	83±13*	87±18*	91±18*	<0.001||
	Female	62±10	77±25	74±19*	67±14	<0.001¶
BMI, kg/m^2^	Male	23.3 ±3.4	25.3±3.7*	27.7±5.3*, ||	27.9±5.5*	<0.001||
	Female	22.6±3.4	26.8±6.4	27.5±6.2*	25.1±4.6*	<0.001¶
Systolic BP, mm Hg	Male	123±9	147±6*	153±11*	133±5*, ||, ¶	<0.001¶
	Female	111±10	146±10*	149±7*	132±6*	<0.001¶
Diastolic BP, mm Hg	Male	73±8	80±6*	97±7*, ||	95±4*, ||	<0.001¶
	Female	71±7	82±7*	102±8*	94±3*	<0.001¶
Pulse pressure, mm Hg	Male	50±9	67±7*	55±10*, ||	39±9*, ||, ¶	<0.001||
	Female	39±8	64±12*	46±7*	38±6||, ¶	<0.001¶
**30–60 y**	**Male**	**n=340**	**n=61**	**n=116**	**n=48**	
	**Female**	**n=661**	**n=61**	**n=78**	**n=45**	
Age, y	Male	45±9	43±11	45±11	47±10	0.26¶
	Female	47±10	54±7*	48±10||	47±10||	<0.001¶
Height, m	Male	1.78±0.07	1.78±0.08	1.78±0.07	1.79±0.09	0.81||
	Female	1.64±0.07	1.62±0.06	1.62±0.08	1.64±0.06	0.05*
Weight, kg	Male	82±13	85±12	89±14*	89±15*	<0.001*
	Female	68±13	74±17*	75±17*	74±16*	<0.001||
BMI, kg/m^2^	Male	26.1±3.8	26.8±3.6	27.9±3.8*	27.8±4.4*	<0.001*
	Female	25.3±4.6	28.4±5.7*	28.3±6.7*	27.8±5.8*	<0.001||
Systolic BP, mm Hg	Male	124±10	147±10*	152±11*	134±5*, ||, ¶	<0.001¶
	Female	116±11	147±7*	153±11*	131±6*, ||, ¶	<0.001¶
Diastolic BP, mm Hg	Male	78±7	84±4*	98±7*, ||	94±3*, ||	<0.001¶
	Female	74±8	83±7*	99±6*, ||	93±3*, ||	<0.001¶
Pulse pressure, mm Hg	Male	46±8	63±11*	54±10*, ||	40±6*, ||, ¶	<0.001||
	Female	41±8	65±11*	54±11*, ||	39±7||, ¶	<0.001||
**>60 y**	**Male**	**n=377**	**n=222**	**n=141**	**n=15**	
	**Female**	**n=580**	**n=286**	**n=107**	**n=19**	
Age, y	Male	68±6	71±6*	68±6||	66±3||	<0.001¶
	Female	67±5	71±6*	69±5||	67±6||	<0.001¶
Height, m	Male	1.74±0.07	1.73±0.07	1.74±0.07	1.74±0.08	0.28*
	Female	1.61±0.06	1.61±0.06	1.61±0.07	1.62±0.06	0.50*
Weight, kg	Male	79±12	82±11*	84±12*	86±14	<0.001*
	Female	67±12	70±14*	69±13	70±13	0.006*
BMI, kg/m^2^	Male	26.0±3.4	27.3±3.6*	27.6±3.7*	28.6±4.4*	<0.001*
	Female	25.7±4.4	27.0±5.3*	26.8±4.2	26.7±4.9	0.001*
Systolic BP, mm Hg	Male	127±8	151±9*	159±14*, ||	135±4||, ¶	<0.001¶
	Female	124±10	151±10*	162±16*	130±7||, ¶	<0.001¶
Diastolic BP, mm Hg	Male	77±7	82±6*	96±6*, ||	93±3*, ||	<0.001¶
	Female	74±7	81±6*	95±5*, ||	93±3*, ||	<0.001¶
Pulse pressure, mm Hg	Male	50±8	69±10*	62±12*, ||	43±5*, ||, ¶	<0.001||
	Female	49±9	70±10*	67±15*	37±7*, ||, ¶	<0.001||

Data are means±SD. BMI indicates body mass index; BP, blood pressure; IDH, isolated diastolic hypertension; ISH, isolated systolic hypertension; and SDH, systolic diastolic hypertension.

* One‐way ANOVA.

^†^ Welch's ANOVA.

^‡^ Kruskall–Wallis test.

^§^ *P*<0.05 vs people without hypertension.

^||^ *P*<0.05 vs ISH.

^¶^ *P*<0.05 vs SDH.

### Hemodynamic Correlates of Hypertensive Phenotypes

Tables 2 shows the hemodynamic characteristics of the 4 BP groups in young men and women. In men with ISH, CO and SV were significantly higher relative to those without hypertension. These differences persisted after adjusting for body size indicating that the increase in SBP was due to a primary elevation in SV rather than secondary to increased body size. In women with ISH, CO was also significantly higher compared with those without hypertension, however, after adjusting for body size the difference was no longer significant. In both men and women with SDH, PVR, aPWV, and AIx were significantly higher relative to those without hypertension. After adjustment for body surface area, the difference in PVR remained significant in both sexes. After adjustment for MAP, the difference in aPWV remained significant only in women and after adjustment for height and heart rate, the difference in AIx remained significant in both sexes. In women with IDH, PVR and AIx were increased significantly compared with those without hypertension, even after adjustment for basic confounders.

**Table 2:** Detailed Hemodynamic Variables According to Blood Pressure Phenotype for Men and Women <30 Years

		Nonhypertensive	ISH	SDH	IDH	*P* value
Cardiac output, L/min	Male	8.4±2.1	9.6±2.1§	8.9±1.8	8.8±2.0	<0.001§
	Female	6.5±1.5	9.3±3.1§	6.9±1.6	6.5±1.3||	0.02||
Cardiac index, L/min/m**	Male	4.3±1.0	4.8±0.9§	4.4±0.9	4.2±1.0	<0.001§
	Female	3.8±0.8	5.1±1.7	3.9±0.7	3.8±0.7	0.12||
Stroke volume, mL	Male	110±30	128±34§	113±27	117±31	<0.001¶
	Female	84±20	116±48	85±22	80±21	0.12||
Stroke volume index, mL/m**	Male	57±14	63±16§	56±14||	56±16	<0.001§
	Female	50±11	64±28	47±11	47±12	0.03¶
Peripheral vascular resistance, dyn s/cm^5^	Male	838±233	786±212	959±191§, ||	900±239	<0.001§
	Female	1032±272	900±319	1343±333§, ||	1275±264§, ||	<0.001¶
Peripheral vascular resistance index, dyn s m^2^/cm^5^	Male	1604±416	1584±413	1952±396§, ||	1887±534||	<0.001§
	Female	1728±423	1648±579	2367±543§, ||	2160±397§, ||	<0.001¶
Aortic PWV, m/s	Male	5.69±0.79	6.02±0.96§	6.90±1.07§, ||	6.35±0.89§	<0.001¶
	Female	5.34±0.71	6.29±0.81§	7.09±1.08§	6.25±0.70§, ¶	<0.001||
Adjusted aortic PWV, m/s#	Male	5.79±0.74	5.76±0.90	6.03±0.93	5.90±0.86	0.38¶
	Female	5.40±0.68	5.73±0.56	5.89±0.99§	5.47±0.62	0.03||
Aortic Aix, %	Male	−2.6±10.6	−4.6±10.7	4.4±12.6§, ||	4.0±14.5||	<0.001§
	Female	2.7±11.5	7.0±12.7	20.7±12.6§, ||	14.9±13.5§	<0.001§
Adjusted aortic Aix, %**	Male	−2.7±10.3	−3.8±10.4	5.2±11.4§, ||	5.1±12.8§, ||	<0.001§
	Female	2.7±11.2	9.8±13.9	20.5±10.4§	15.5±11.9§	<0.001¶
Mean arterial pressure, mm Hg	Male	82±7	90±8§	103±8§, ||	94±7§, ¶	<0.001||
	Female	79±7	95±9§	111±10§, ||	100±7§, ¶	<0.001||
Heart rate, beats/min	Male	65±11	68±12	72±12§	70±13	0.002||
	Female	67±11	78±11§	74±12§	74±11§	<0.001§

Data are means±SD. AIx indicates augmentation index; IDH, isolated diastolic hypertension; ISH, isolated systolic hypertension; PWV, pulse wave velocity; and SDH, systolic‐diastolic hypertension.

* One‐way ANOVA.

^†^ Welch's ANOVA.

^‡^ Kruskall–Wallis test.

^§^ *P*<0.05 vs people without hypertension.

^||^ *P*<0.05 vs ISH.

^¶^ *P*<0.05 vs SDH.

^#^ Data adjusted for mean arterial pressure.

** Data adjusted for height and heart rate.

In the middle age group, the overall hemodynamic patterns presented in Table 3 tended to be more mixed. Men with ISH had increased CO and CI compared with those without hypertension, but their SV was similar to the group without hypertension. In contrast, women with ISH had increased PVR, aPWV, and AIx relative to those without hypertension. Adjustments for basic confounders did not change these results. Men and women with SDH were characterized by increased PVR, aPWV, and AIx relative to those without hypertension, and after adjustment for basic confounders, the results did not change in relation to PVR and Aix; however, the difference in adjusted aPWV was no longer significant. Furthermore, women with SDH had elevated CO relative to those without hypertension. While men with IDH were characterized by a decreased SVI and an increased PVRI compared with those without hypertension, women with IDH showed increased PVR, PVRI, aPWV, and adjusted AIx relative to those without hypertension.

**Table 3:** Detailed Hemodynamic Variables According to Blood Pressure Phenotype for Men and Women 30 to 60 Years

		Nonhypertensive	ISH	SDH	IDH	*P* value
Cardiac output, L/min	Male	6.6±1.9	7.6±1.8§	7.0±1.8	6.6±1.8||	<0.001§
	Female	5.5±1.5	5.5±1.6	6.0±1.7§	5.6±1.7	0.06§
Cardiac index, L/min/m^2^	Male	3.3±0.9	3.7±0.8§	3.4±0.8	3.2±0.9||	0.003§
	Female	3.2±0.8	3.1±0.8	3.4±1.0	3.1±0.9	0.17§
Stroke volume, mL	Male	102±29	108±26	97±24	93±28||	0.02§
	Female	81±23	75±23	79±23	78±23	0.26§
Stroke volume index, mL/m^2^	Male	51±14	53±12	47±11§, ||	45±14§, ||	0.001||
	Female	47±13	42±12§	45±13	44±13	0.02§
Peripheral vascular resistance, dyn s/cm^5^	Male	1166±378	1058±311	1324±472§, ||	1317±442||	<0.001§
	Female	1336±391	1619±515§	1630±649§	1515±395§	<0.001||
Peripheral vascular resistance index, dyn s m^2^/cm^5^	Male	2315±727	2121±576	2711±886§, ||	2710±880§, ||	<0.001§
	Female	2301±663	2872±897§	2893±1052§	2692±591§	<0.001||
Aortic PWV, m/s	Male	7.03±1.31	7.24±1.57	7.94±1.56§, ||	7.56±1.12	<0.001§
	Female	6.74±1.35	8.61±1.65§	8.19±1.90§	7.49±1.29§, ||	<0.001||
Adjusted aortic PWV, m/s#	Male	7.31±1.22	7.15±1.54	7.26±1.52	7.27±1.07	0.85§
	Female	7.01±1.23	7.82±1.69§	6.83±1.78||	6.87±1.34||	0.003||
Aortic Aix, %	Male	14.7±10.7	13.3±11.7	19.2±10.4§, ||	14.8±12.4	0.001§
	Female	26.7±10.5	30.9±9.1§	31.0±9.6§	29.9±7.9	<0.001§
Adjusted aortic Aix, %**	Male	14.2±10.0	13.7±10.1	20.1±8.9§, ||	16.2±12.0	<0.001§
	Female	26.4±9.7	31.9±7.6§	31.9±7.9§	30.8±6.9§	<0.001||
Mean arterial pressure, mm Hg	Male	89±7	96±7§	108±10§, ||	99±6§, ¶	<0.001||
	Female	86±8	102±8§	111±10§, ||	99±6§, ¶	<0.001§
Heart rate, beats/min	Male	63±10	65±12	67±11§	68±12§	<0.001§
	Female	65±9	70±11§	70±10§	68±10	<0.001§

Data are means±SD. AIx indicates augmentation index; IDH, isolated diastolic hypertension; ISH, isolated systolic hypertension; SDH, systolic diastolic hypertension; and PWV, pulse wave velocity.

* One‐way ANOVA.

^†^ Welch's ANOVA.

^‡^ Kruskall–Wallis test.

^§^ *P*<0.05 vs people without hypertension.

^||^ *P*<0.05 vs ISH.

^¶^ *P*<0.05 vs SDH.

^#^ Data adjusted for mean arterial pressure.

** Data adjusted for height and heart rate.

Table 4 shows the hemodynamic characteristics of the BP phenotypes in older individuals. No differences between groups were observed in either CO or SV in men or women. However, in contrast to ISH in young individuals, older men and women with ISH were characterized by elevated PVR and aPWV compared with those without hypertension, which persisted after adjusting for basic confounders. In addition, adjusted AIx was also increased both in men and women with ISH relative to individuals without hypertension. In men and women with SDH, PVR, PVRI, and adjusted AIx were elevated compared with those without hypertension. Both men and women with IDH had the lowest aPWV among the groups with hypertension.

**Table 4:** Detailed Hemodynamic Variables According to Blood Pressure Phenotype for Men and Women >60 Years

		Nonhypertensive	ISH	SDH	IDH	*P* value
Cardiac output, L/min	Male	5.3±1.6	5.4±1.3	5.7±1.7	5.1±1.3	0.10§
	Female	4.5±1.3	4.7±1.2	4.7±1.4	4.7±1.2	0.15§
Cardiac index, L/min/m^2^	Male	2.8±0.8	2.8±0.7	2.9±0.8	2.5±0.6	0.33§
	Female	2.7±0.7	2.8±0.7	2.8±0.8	2.7±0.6	0.48§
Stroke volume, mL	Male	82±27	81±23	82±26	72±21	0.54§
	Female	67±19	67±18	64±16	62±18	0.16§
Stroke volume index, mL/m**	Male	42±13	42±11	41±13	36±10	0.24§
	Female	40±11	39±10	37±9	36±10	0.07§
Peripheral vascular resistance, dyn s/cm^5^	Male	1506±492	1606±471§	1700±540§	1671±472	<0.001¶
	Female	1701±511	1820±501§	2052±656§, ||	1810±466	<0.001||
Peripheral vascular resistance index, dyn s m^2^/cm^5^	Male	2894±919	3120±895§	3352±1022§	3319±783	<0.001§
	Female	2881±864	3122±833§	3517±1157§, ||	3130±746	<0.001||
Aortic PWV, m/s	Male	8.68±2.06	10.08±2.47§	10.16±2.54§	8.52±1.17||, ¶	<0.001||
	Female	8.23±1.88	9.78±2.22§	10.37±2.39§	8.35±1.43||, ¶	<0.001||
Adjusted aortic PWV, m/s#	Male	9.21±2.00	9.85±2.46§	9.14±2.35||	8.42±1.07||	<0.001||
	Female	8.69±1.83	9.35±2.21§	9.13±2.37	7.96±1.35||, ¶	<0.001||
Aortic Aix, %	Male	25.5±8.4	26.9±8.3	27.2±7.7	24.9±7.4	0.09§
	Female	32.6±8.1	33.5±8.3	34.0±7.7	33.5±6.1	0.27§
Adjusted aortic Aix, %**	Male	25.1±7.2	26.7±7.1§	28.4±6.8§	27.7±5.1	<0.001¶
	Female	32.2±7.0	33.8±6.5§	35.5±5.5§	35.3±5.0	<0.001¶
Mean arterial pressure, mm Hg	Male	91±7	101±8§	111±10§, ||	99±4§, ¶	<0.001||
	Female	90±8	101±8§	112±9§, ||	101±8§, ¶	<0.001§
Heart rate, beats/min	Male	64±10	65±10	68±11§	72±12§	<0.001§
	Female	66±9	68±10§	71±12§, ||	71±8	<0.001§

Data are means±SD. AIx indicates augmentation index; IDH, isolated diastolic hypertension; ISH, isolated systolic hypertension; PWV, pulse wave velocity; and SDH, systolic diastolic hypertension.

* One‐way ANOVA.

^†^ Welch's ANOVA.

^‡^ Kruskall–Wallis test.

^§^ *P*<0.05 vs people without hypertension.

^||^ *P*<0.05 vs ISH.

^¶^ *P*<0.05 vs SDH.

^#^ Data adjusted for mean arterial pressure.

** Data adjusted for height and heart rate.

**Figure 2:**
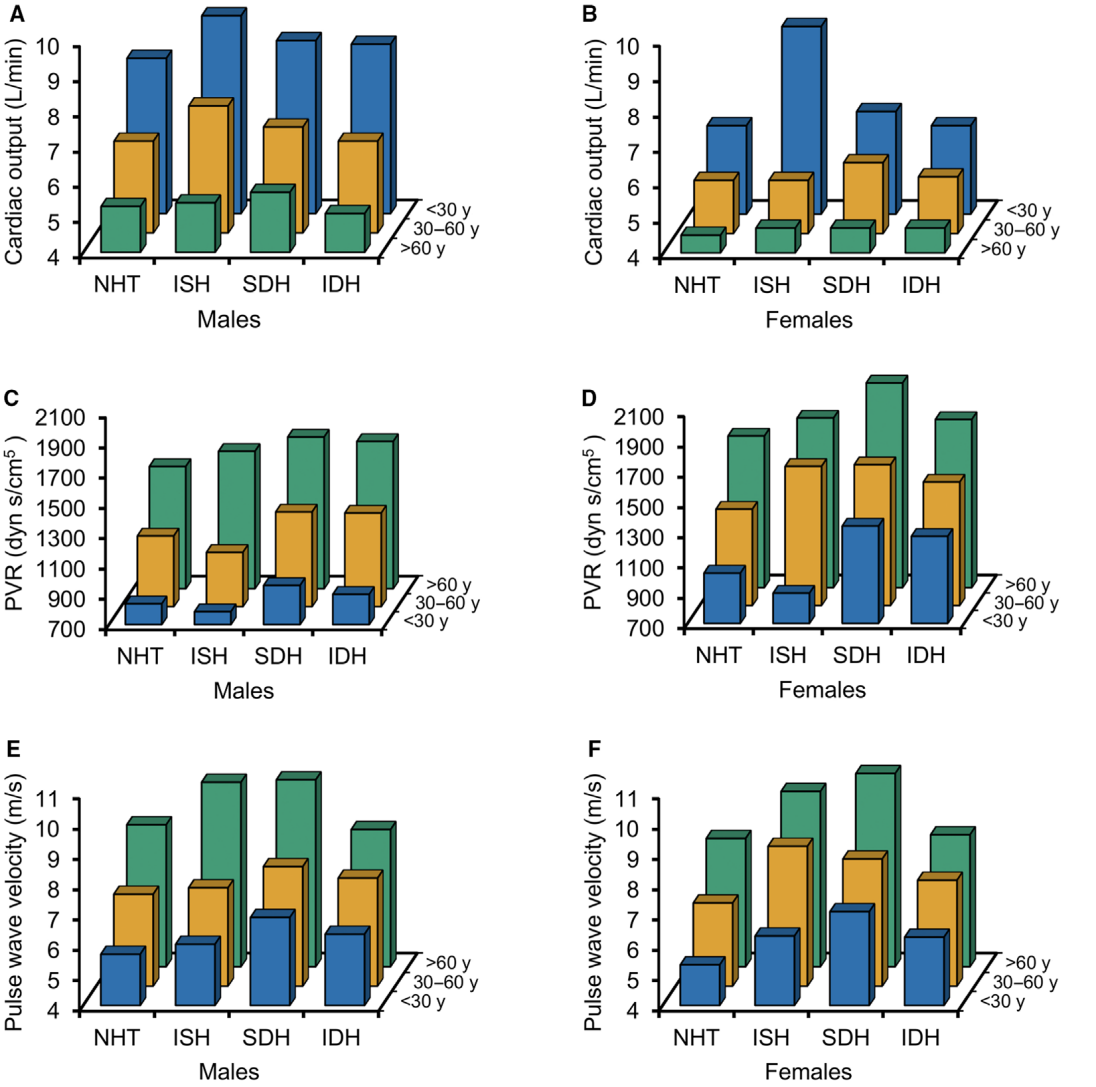
Hemodynamic variables according to blood pressure phenotype, stratified by age and sex. Cardiac output in men (**A**) and women (**B**), peripheral vascular resistance in men (**C**) and women (**D**), and pulse wave velocity in men (**E**) and women (**F**). Data are means. IDH indicates isolated diastolic hypertension; ISH, isolated systolic hypertension; NHT, nonhypertensive; PVR, peripheral vascular resistance; and SDH, systolic diastolic hypertension.

Figure 2 illustrates the major hemodynamic variables, CO (Figure 2A and 2B), PVR (Figure 2C and 2D), and aPWV (Figure 2E and 2F), according to BP phenotype, stratified by age and sex.

Tables S2–S5 show the results of our exploratory analyses. Table S2 shows that participants with prehypertension had increased BMI compared with individuals with optimal BP in the young and middle‐aged groups. In young individuals, the hemodynamic patterns in ISH, SDH, and IDH compared with individuals with optimal BP were very similar to those presented in our main analysis (Table S3). Interestingly, both men and women with prehypertension had increased CO and CI compared with the optimal BP group. Table S4 did not reveal new details about the hemodynamic patterns of hypertensive phenotypes in middle‐aged adults; however, we observed increased PVR and PVRI in female individuals with prehypertension compared with women with optimal BP. Table S5 confirmed the results of our main analysis that increased aPWV is the main alteration in older men with ISH. Similarly to the middle‐aged group, female individuals with prehypertension had increased PVR and PVRI compared with individuals with optimal BP.

## DISCUSSION

We have demonstrated that essential hypertension is characterized by distinct phenotypes, which vary across the adult age span. Significant sex‐related differences in the hemodynamic mechanisms underlying these phenotypes are also apparent, especially in younger adults, underpinning a need to reconsider how hypertension is treated and whether this should be similar between men and women.

The arterial BP has 2 major physiological components, besides the simple extremes of SBP and DBP. The steady state component, represented by the MAP, is determined by CO and PVR. In contrast, the pulsatile component, represented by the pulse pressure, is determined mainly by SV and aortic stiffness.^23^ Derangements in one or more of the determinants of BP could directly contribute to BP elevation and the development of essential hypertension. Two recent analyses focused on hemodynamic mechanisms underlying hypertension using large sample sizes.^24^, ^25^ However, the generalizability of the findings is limited. Aristizábal‐Ocampo et al. used 24‐hour ambulatory BP monitoring and estimated hemodynamic parameters based on a two‐element Windkessel model in 7473 subjects. However, hemodynamic parameters were only indirectly estimated and results were not stratified by age and sex.^24^ Yano et al. examined left ventricular SV, systemic vascular resistance, and aortic arch PWV using cardiovascular magnetic resonance imaging across different hypertensive phenotypes in a relatively large population of the Dallas Heart Study (n=2001).^26^ However, their results were presented using broad age ranges (18–49 years and 18–64 years), and their data were not stratified by sex. Li et al. performed a meta‐analysis of 27 studies (n=11 765). However, CO was measured using 7 different techniques, aortic stiffness was only determined in 11 studies using heterogenous methods, only 3 studies provided separate data for men and women, a few studies included solely male participants, and hypertensive phenotypes were defined in only 2 studies. Furthermore, the results of the meta‐analysis were provided only for 2 age groups (children and young adults [<35 years]; older adults [≥35 years]).^25^ Our study is the first to examine the major hemodynamic mechanisms behind different hypertensive phenotypes across different age groups and sexes using consistent methods in a sufficiently large population.

Previous epidemiological studies in young male adults and adolescents have confirmed that ISH is the most common form of hypertension in this group.^11^, ^27^, ^28^ As expected, the current study confirmed the preponderance of ISH over SDH in men aged <30 years, and the condition predominantly affected men rather than women (~12:1) within this age group. Higher BMI, CO, and SV were observed in young men with ISH, and adjusting CO and SV for body size did not change our results. A high output‐low resistance circulatory pattern was previously observed in a small (n=32) group of young men with high brachial pulse pressure,^29^ and the aforementioned meta‐analysis showed that in children and young adults with hypertension, elevated CO is the primary hemodynamic alteration,^25^ in line with our results. In agreement with these findings, our exploratory analysis revealed increased CO and CI in young men with prehypertension, suggesting that the high output‐low resistance pattern is already present in a significant portion of individuals at the prehypertensive stage. Yano et al. did not observe increased SV in patients with ISH.^26^ One potential explanation is that the population of the Dallas Heart Study was more diverse (~50% of Black racial origin),^30^ compared with our study. The Bogalusa Heart Study showed that in young adults, elevation of CO is the dominant factor in the development of hypertension in White individuals, whereas increased PVR is dominant in Black individuals.^31^ This could explain the absence of difference in SV between individuals with ISH and individuals with optimal BP, and the increased PVR in participants with ISH in the Dallas Heart Study. In contrast to young men with ISH, young men with SDH were characterized by increased PVR and PVRI.

Interestingly, far fewer studies examined the specific hemodynamic mechanisms underlying hypertension in women. A previous analysis of The Enigma Study showed that a vascular phenotype, characterized by elevated PVR, increased wave reflections, and increased arterial stiffness, is associated with the development of essential hypertension in young women.^32^ Our findings extend these observations by providing, for the first time, a detailed insight into different hypertensive phenotypes in young women. In contrast to men, SDH and IDH were more common than ISH in younger women, and these phenotypes were primarily associated with elevated PVR and AIx. These patterns remained independent of confounding factors. Furthermore, SDH was also associated with increased aPWV, even after adjustment for MAP. The fact that such marked differences exist between men and women at such a relatively young age suggests very different causal mechanisms between the sexes, arguing against essential hypertension being treated as a uniform condition and in support of the need for different therapeutic approaches.

Middle‐aged individuals portrayed a complex picture. While the main hemodynamic disturbance was increased CO in middle‐aged men, middle‐aged women with ISH were characterized by a consistent vascular phenotype of increased PVR, aortic stiffness, and wave reflections. However, SDH was the predominant form of hypertension in both men and women and characterized by increased PVR. Although BMI was higher relative to individuals without hypertension in both sexes, adjustment for body size did not change these results. The SDH phenotype was also associated with higher aPWV, although this was attenuated after adjustment for MAP, suggesting that structural alterations of the aortic wall do not play a major role in the development of this phenotype. Our cross‐sectional observations concerning the dominant hypertensive phenotypes in young and middle‐aged individuals support earlier longitudinal observations, albeit in small numbers, which suggest that the early phase of hypertension is characterized by a hyperdynamic circulation, which transforms over time to a normalized output‐high resistance circulation, most likely due to resistance vessel remodeling, and the subsequent development of sustained hypertension.^33^ As such, early, targeted treatments to delay any structural alterations in resistance vessels are likely to be beneficial. Interestingly, our exploratory analysis suggests that resistance vessel remodeling may already begin in middle‐aged women with prehypertension. A recent large (n=34 238) study performed by Mahajan et al. examined CO (and CI) and PVR (and PVRI) using impedance cardiography in mainly middle‐aged individuals grouped by brachial SBP.^34^ Among individuals with SBP ≥140 mm Hg, 12 473 (73%) patients had increased PVRI and normal/low CI, and 2370 (14%) patients had increased CI and normal/low PVRI. In our study, increased PVR (and PVRI) was the dominant alteration in middle‐aged men and women with SDH and women with ISH, and increased CO (and CI) was the dominant mechanism in middle‐aged men with ISH. Although Mahajan et al. looked only at static BP components, and used a high‐throughput clinical setting across multiple centres, we observed similar patterns. However, importantly, our study also focussed on pulsatile BP components and adds further information about the age‐ and sex‐specific alterations. Furthermore, information about DBP is also provided in our study.

In addition to being the predominant form of hypertension in younger individuals, ISH was also the predominant form of hypertension in older individuals, with two key differences. First, there was no preponderance of men over women with ISH. Second, the hemodynamic mechanisms were markedly different. ISH in older adults was characterized by an elevated aPWV, even after adjustment for MAP, in both men and women, confirming previous reports that ISH in older individuals is characterized by excessive aortic stiffening.^35^, ^36^ Interestingly, PVR was also slightly, but significantly higher in older individuals with ISH versus those without hypertension. Longitudinal data from the Framingham Heart Study showed that the majority of individuals (59%) with new‐onset ISH did not have antecedent diastolic hypertension, therefore, the dominant hemodynamic mechanism of the development of ISH is aortic stiffening.^37^ However, in a minority of patients, large‐artery stiffening could have superimposed on antecedent increased PVR, which may, in part, explain the higher PVR, on average, in subjects with ISH in the current study. Our exploratory analysis confirmed that increased aortic stiffness is the dominant hemodynamic mechanism underlying ISH in older men, and increased aortic stiffness together with increased PVR and PVRI is associated with ISH in older women. In older individuals with SDH, the substantially increased PVR explained the increased SBP and DBP. Increased PVR and PVRI, observed in older women with ISH or SDH, are also evident in older prehypertensive women. This suggests that alterations in the peripheral vasculature may already be present at the prehypertensive stage. In the small number of older idividuals with IDH, we could not observe substantial and characteristic hemodynamic alterations.

There are several limitations of our study. The current study has a cross‐sectional design, and, although longitudinal data from large cohort studies such as the Framingham Heart Study are available, comprehensive hemodynamic assessments including CO and PVR have not been undertaken systematically. Therefore, longitudinal follow‐up of healthy individuals is clearly required to confirm our observations and provide evidence of underlying causal mechanisms, which may ultimately aid in timely intervention and prevention of future sustained hypertension. The population was of predominantly White racial origin, and the examination of racially and ethnically more diverse populations could provide additional valuable insights. Our results should be interpreted with caution for certain subgroups, such as young men with IDH or young women with ISH, due to the small sample size. In addition, we excluded individuals with certain comorbidities such as type 2 diabetes or hypercholesterolemia to avoid their potentially confounding effects in the analyses. Nevertheless, these exclusions may limit the generalizability of our results. Also, BP phenotypes were defined based on a single office visit. Additionally, lifestyle factors like physical activity and exercise were not considered in these analyses. As high levels of physical activity are associated with both increased CO and also lower aPWV and AIx, understanding the influence of physical activity on these hypertensive phenotypes may provide important additional information.

### Clinical Relevance

Although average BP levels of the general population and people treated for hypertension have progressively improved over previous decades,^3^ BP remains poorly controlled in a substantial number of patients due to various reasons such as disparities in access to care or medication adherence.^3^, ^6^, ^38^, ^39^ Another important factor might be that essential hypertension is often viewed and treated as a uniform condition or following protocols simply based on age.^40^ Our results highlight the potential for a more nuanced approach and offer an initial step toward protocols that also take account of hypertensive phenotypes and the sex‐specific hemodynamic mechanisms underlying these. Since essential hypertension, once established, is treatable but not curable, and BP tracks over the life course,^9^, ^41^ early interventions targeted toward the age‐, sex‐, and phenotype‐specific hemodynamic mechanisms described in our article are likely to be key to enhancing BP control. Previous results suggest that individual noninvasive hemodynamic profiling can improve hypertension management.^42^, ^43^, ^44^, ^45^ However, these techniques are not currently available in everyday clinical practice due to financial and logistic reasons. Until comprehensive hemodynamic profiling of each individual is feasible and widely accessible, targeting the existing therapeutic repertoire toward the phenotype‐specific dominant hemodynamic alterations presented in our work may represent the next step toward more effective hypertension management.

## Conclusions

In summary, our study provides important evidence that essential hypertension is a highly heterogeneous condition and the hemodynamic mechanisms behind different hypertensive phenotypes differ significantly between sexes and also change across the adult age span.

## Sources of Funding

Ian B. Wilkinson was supported by a British Heart Foundation Senior Clinical Fellowship and Carmel M. McEniery by a British Heart Foundation Intermediate Research Fellowship. Carmel M. McEniery was also supported by the National Institute for Health Research Cambridge Biomedical Research Centre (BRC‐1215‐20 014). This work was funded in part through the British Heart Foundation and the National Institute for Health Research Cambridge Biomedical Research Centre. The views expressed are those of the authors and not necessarily those of the National Institute for Health Research or the Department of Health and Social Care.

## Disclosures

Kaisa M. Mäki‐Petäjä was employed at University of Cambridge when the study was conducted but now works at AstraZeneca, Cambridge, UK. The other authors have nothing to declare.

## Supporting information

Tables S1–S5

## Appendix

The ACCT Investigators include John R. Cockcroft, Zahid Dhakam, Stacey S. Hickson, Julia Howard, Kaisa M. Mäki‐Petäjä, Barry J. McDonnell, Carmel M. McEniery, Karen Miles, Maggie Munnery, Pawan Pusalkar, Christopher Retallick, Jane Smith, Edna Thomas, Sharon Wallace, Ian B. Wilkinson, Susannah Williams, Jean Woodcock‐Smith and Yasmin.
