# Correction to: Phenotypes of Hypertension: Impact of Age and Sex on Hemodynamic Mechanisms

**DOI:** 10.1161/JAHA.125.045297

**Published:** 2025-11-11

In the article by Cseh et al, “Phenotypes of Hypertension: Impact of Age and Sex on Hemodynamic Mechanisms” (*J Am Heart Assoc*. 2025;14:e042096. DOI: 10.1161/JAHA.125.042096), which was published on August 29, 2025 and appeared in the September 2, 2025 issue of the Journal, corrections were needed.

In the production process, errors were introduced into the symbols and footnotes of Tables 1–4. These symbols and footnotes have now been corrected while retaining the authors’ original meaning.

The publisher regrets the error.

The online version of the article has been updated and is available at https://www.ahajournals.org/doi/full/10.1161/JAHA.125.042096.

