# Supplementary material for: Phenotypes of Hypertension: Impact of Age and Sex on Hemodynamic Mechanisms
Source: J Am Heart Assoc. 2025 Aug 29;14(17):e042096. doi: 10.1161/JAHA.125.042096 (PMC12553408; doi:10.1161/JAHA.125.042096)
Supplement: Supplementary file 1 — Tables S1–S5 [file JAH3-14-e042096-s001.pdf]

Supplemental Materials

Table S1A. Distribution of individuals in the study population according to blood pressure phenotype, stratified by age and sex.

	NHT		ISH		SDH		IDH		Total
**Overall**	**n**	**% within age range**	**n**	**% within age range**	**n**	**% within age range**	**n**	**% within age range**	**n**
<30 years	1930	87.2	150	6.8	88	4.0	46	2.1	2214
30-60 years	1001	71.0	122	8.7	194	13.8	93	6.6	1410
>60 years	957	54.8	508	29.1	248	14.2	34	1.9	1747
Total	3888	72.4	780	14.5	530	9.9	173	3.2	5371
**Males**	**n**	**% within age range**	**n**	**% within age range**	**n**	**% within age range**	**n**	**% within age range**	**n**
<30 years	874	80.8	138	12.8	55	5.1	15	1.4	1082
30-60 years	340	60.2	61	10.8	116	20.5	48	8.5	565
>60 years	377	49.9	222	29.4	141	18.7	15	2.0	755
Total	1591	66.2	421	17.5	312	13.0	78	3.2	2402
**Females**	**n**	**% within age range**	**n**	**% within age range**	**n**	**% within age range**	**n**	**% within age range**	**n**
<30 years	1056	93.3	12	1.1	33	2.9	31	2.7	1132
30-60 years	661	78.2	61	7.2	78	9.2	45	5.3	845
>60 years	580	58.5	286	28.8	107	10.8	19	1.9	992
Total	2297	77.4	359	12.1	218	7.3	95	3.2	2969

IDH=isolated diastolic hypertension; ISH=isolated systolic hypertension; NHT=non-hypertensive; SDH=systolic diastolic hypertension

Table S1B. Distribution of hypertensive individuals in the study population according to hypertensive phenotype, stratified by age and sex.

	ISH		SDH		IDH		Total	
**Overall**	**n**	**% within age range**	**n**	**% within age range**	**n**	**% within age range**	**n**	
<30 years	150	52.8	88	31.0	46	16.2	284	
30-60 years	122	29.8	194	47.4	93	22.7	409	
>60 years	508	64.3	248	31.4	34	4.3	790	
**Males**	**Observed n**	**Expected n**	**Observed n**	**Expected n**	**Observed n**	**Expected n**	**n**	**P¶**
<30 years	138	110	55	64	15	34	208	
30-60 years	61	67	116	107	48	51	225	<0.001
>60 years	222	243	141	119	15	16	378	
**Females**	**Observed n**	**Expected n**	**Observed n**	**Expected n**	**Observed n**	**Expected n**	**n**	**P§**
<30 years	12	40	33	24	31	12	76	
30-60 years	61	55	78	87	45	42	184	<0.001
>60 years	286	265	107	129	19	18	412	

P¶ chi-squared goodness-of-fit test, males vs overall population

P§ chi-squared goodness-of-fit test, females vs overall population

IDH=isolated diastolic hypertension; ISH=isolated systolic hypertension; NHT=non-hypertensive; SDH=systolic diastolic hypertension

Table S2. Anthropometric and seated blood pressure data across five blood pressure phenotypes, stratified by age and sex.

		Optimal BP	Prehypertension	ISH	SDH	IDH	P
**<30 years**	**M**	**n=291**	**n=583**	**n=138**	**n=55**	**n=15**	
	**F**	**n=792**	**n=264**	**n=12**	**n=33**	**n=31**	
Age (years)	M	21±2	21±3	21±3	23±4*#†	23±4	<0.001[c]
	F	21±3	21±3	21±3	24±4*#	22±3	<0.001[c]
Height (m)	M	1.78±0.07	1.80±0.07*	1.81±0.07*	1.77±0.08†	1.80±0.05	<0.001[a]
	F	1.66±0.07	1.67±0.07	1.68±0.09	1.63±0.06	1.62±0.05#	0.006[a]
Weight (kg)	M	71±11	77±12*	83±13*#	87±18*#	91±18*	<0.001[b]
	F	61±10	65±11*	77±25	74±19*	67±14	<0.001[c]
BMI (kg/m^2)	M	22.3±3.0	23.9±3.5*	25.3±3.7*#	27.7±5.3*#†	27.9±5.5*	<0.001[b]
	F	22.3±3.2	23.7±3.8*	26.8±6.4	27.5±6.2*#	25.1±4.6*	<0.001[c]
Systolic BP (mmHg)	M	112±6	128±6*	147±6*#	153±11*#	133±5*†§	<0.001[c]
	F	107±8	122±7*	146±10*	149±7*#	132±6*	<0.001[c]
Diastolic BP (mmHg)	M	68±6	75±7*	80±6*#	97±7*#†	95±4*#†	<0.001[c]
	F	69±6	79±6*	82±7*	102±8*#	94±3*#	<0.001[c]
Pulse pressure (mmHg)	M	44±7	53±9*	67±7*#	55±10*†	39±9#†§	<0.001[b]
	F	38±7	43±10*	64±12*#	46±7*	38±6#†§	<0.001[c]
**30-60 years**	**M**	**n=94**	**n=246**	**n=61**	**n=116**	**n=48**	
	**F**	**n=369**	**n=292**	**n=61**	**n=78**	**n=45**	
Age (years)	M	44±10	46±9	43±11	45±11	47±10	0.090[c]
	F	46±10	49±9*	54±7*#	48±10†	47±10†	<0.001[c]
Height (m)	M	1.77±0.06	1.78±0.07	1.78±0.08	1.78±0.07	1.79±0.09	0.503[b]
	F	1.64±0.07	1.63±0.06	1.62±0.06	1.62±0.08	1.64±0.06	0.056[a]
Weight (kg)	M	77±11	84±13*	85±12*	89±14*#	89±15*	<0.001[a]
	F	65±11	71±14*	74±17*	75±17*	74±16*	<0.001[b]
BMI (kg/m^2)	M	24.7±3.4	26.6±3.8*	26.8±3.6*	27.9±3.8*#	27.8±4.4*	<0.001[a]
	F	24.2±3.8	26.7±5.2*	28.4±5.7*	28.3±6.7*	27.8±5.8*	<0.001[b]
Systolic BP (mmHg)	M	112±6	128±6*	147±10*#	152±11*#	134±5*#†§	<0.001[c]
	F	108±7	126±6*	147±7*#	153±11*#	131±6*†§	<0.001[c]
Diastolic BP (mmHg)	M	71±5	81±5*	84±4*#	98±7*#†	94±3*#†	<0.001[c]

	F	70±6	80±6*	83±7*	99±6*#†	93±3*#†	<0.001[c]
Pulse pressure (mmHg)	M	41±6	48±7*	63±11*#	54±10*#†	40±6#†§	<0.001[b]
	F	38±6	46±8*	65±11*#	54±11*#†	39±7#†§	<0.001[b]
**>60 years**	**M**	**n=60**	**n=317**	**n=222**	**n=141**	**n=15**	
	**F**	**n=171**	**n=409**	**n=286**	**n=107**	**n=19**	
Age (years)	M	68±6	69±6	71±6*#	68±6†	66±3†	<0.001[c]
	F	67±5	68±5	71±6*#	69±5*†	67±6†	<0.001[c]
Height (m)	M	1.75±0.08	1.74±0.07	1.73±0.07	1.74±0.07	1.74±0.08	0.324[a]
	F	1.61±0.06	1.61±0.06	1.61±0.06	1.61±0.07	1.62±0.06	0.661[a]
Weight (kg)	M	77±12	79±11	82±11*	84±12*#	86±14*	<0.001[a]
	F	64±11	68±12	70±14*	69±13*	70±13	<0.001[a]
BMI (kg/m^2)	M	25.1±3.2	26.2±3.4	27.3±3.6*#	27.6±3.7*#	28.6±4.4*	<0.001[a]
	F	24.9±4.3	26.0±4.4	27.0±5.3*	26.8±4.2*	26.7±4.9	<0.001[a]
Systolic BP (mmHg)	M	114±5	130±6*	151±9*#	159±14*#†	135±4*†§	<0.001[c]
	F	111±7	129±6*	151±10*#	162±16*#	130±7*†§	<0.001[c]
Diastolic BP (mmHg)	M	71±6	78±7*	82±6*#	96±6*#†	93±3*#†	<0.001[c]
	F	69±6	77±6*	81±6*#	95±5*#†	93±3*#†	<0.001[c]
Pulse pressure (mmHg)	M	42±6	51±8*	69±10*#	62±12*#†	43±5#†§	<0.001[b]
	F	42±7	53±8*	70±10*#	67±15*#†	37±7#†§	<0.001[b]

Data are means ± SD.

[a]One-way ANOVA, [b]Welch's ANOVA, [c]Kruskall-Wallis test.

*P<0.05 versus optimal BP, #P<0.05 versus prehypertension, †P<0.05 versus ISH, §P<0.05 versus SDH

BMI=body mass index; BP=blood pressure; F=female; IDH=isolated diastolic hypertension; ISH=isolated systolic hypertension; M=Male; SDH=systolic diastolic hypertension.

Table S3. Detailed haemodynamic variables across five blood pressure phenotypes for males and females <30 years

		Optimal BP	Prehypertension	ISH	SDH	IDH	P
**Cardiac output (L/min)**	M	7.7±1.9#	8.7±2.1*	9.6±2.1*#	8.9±1.8*	8.8±2.0	<0.001[a]
	F	6.3±1.5	6.9±1.6*	9.3±3.1*	6.9±1.6	6.5±1.3	<0.001[b]
**Cardiac index (L/min/m²)**	M	4.1±0.9	4.5±1.0*	4.8±0.9*#	4.4±0.9	4.2±1.0	<0.001[a]
	F	3.8±0.8	4.0±0.9*	5.1±1.7	3.9±0.7	3.8±0.7	<0.001[b]
**Stroke volume (mL)**	M	104±29	113±31*	128±34*#	113±27	117±31	<0.001[c]
	F	84±20	84±20	116±48	85±22	80±21	0.191[b]
**Stroke volume index (mL/m²)**	M	55±14	57±15	63±16*#	56±14†	56±16	<0.001[c]
	F	50±11	49±11	64±28	47±11	47±12	0.012[c]
**Peripheral vascular resistance (dyn s/cm⁵)**	M	869±244	823±226	786±212*	959±191#†	900±239	<0.001[a]
	F	1026±260	1049±306	900±319	1343±333*#†	1275±264*#†	<0.001[c]
**Peripheral vascular resistance index (dyn s m²/cm⁵)**	M	1615±409	1599±419	1584±413	1952±396*#†	1887±534	<0.001[a]
	F	1706±398	1794±485	1648±579	2367±543*#†	2160±397*#†	<0.001[c]
**Aortic PWV (m/s)**	M	5.51±0.74	5.78±0.79*	6.02±0.96*	6.90±1.07*#†	6.35±0.89*	<0.001[c]
	F	5.25±0.68	5.61±0.71*	6.29±0.81*	7.09±1.08*#	6.25±0.70*#§	<0.001[b]
**Adjusted aortic PWV (m/s)[1]**	M	5.81±0.72	5.79±0.75	5.76±0.90	6.03±0.93	5.90±0.86	0.508[a]
	F	5.40±0.68	5.41±0.68	5.73±0.56	5.89±0.99*#	5.47±0.62	0.043[a]
**Aortic Aix (%)**	M	-1.0±10.5	-3.4±10.5*	-4.6±10.7*	4.4±12.6*#†	4.0±14.5†	<0.001[a]
	F	2.4±11.5	3.6±11.6	7.0±12.7	20.7±12.6*#†	14.9±13.5*#	<0.001[a]
**Adjusted aortic AIx (%)[2]**	M	-1.5±10.6	-3.3±10.1	-3.8±10.4	5.2±11.4*#†	5.1±12.8#†	<0.001[a]
	F	2.2±11.2	4.3±10.8	9.8±13.9	20.5±10.4*#	15.5±11.9*#	<0.001[c]
**Mean arterial pressure (mmHg)**	M	78±6	84±6*	90±8*#	103±8*#†	94±7*#§	<0.001[b]
	F	77±6	86±7*	95±9*#	111±10*#†	100±7*#§	<0.001[b]
**Heart rate (beats/min)**	M	64±11	66±11*	68±12*	72±12*#	70±13	<0.001[a]
	F	66±11	71±11*	78±11*#	74±12*#†	74±11*#§	<0.001[b]

Data are means ± SD.

[a]One-way ANOVA, [b]Welch's ANOVA, [c]Kruskal-Wallis test.

*P<0.05 versus optimal BP, #P<0.05 versus prehypertension, †P<0.05 versus ISH, §P<0.05 versus SDH

[1]Data adjusted for mean arterial pressure. [2]Data adjusted for height and heart rate.

AIx=augmentation index; BP=blood pressure; F=female; IDH=isolated diastolic hypertension; ISH=isolated systolic hypertension; M=Male; SDH=systolic-diastolic hypertension; PWV=pulse wave velocity.

Table S4. Detailed haemodynamic variables across five blood pressure phenotypes for males and females 30-60 years

		Optimal BP	Prehypertension	ISH	SDH	IDH	P
**Cardiac output (L/min)**	M	6.5±1.8	6.7±1.9	7.6±1.8*#	7.0±1.8	6.6±1.8†	0.002[a]
	F	5.4±1.4	5.6±1.5	5.5±1.6	6.0±1.7*	5.6±1.7	0.041[a]
**Cardiac index (L/min/m²)**	M	3.3±1.0	3.3±0.9	3.7±0.8#	3.4±0.8	3.2±0.9†	0.008[a]
	F	3.2±0.8	3.2±0.9	3.1±0.8	3.4±1.0	3.1±0.9	0.282[a]
**Stroke volume (mL)**	M	101±31	102±28	108±26	97±24	93±28	0.043[a]
	F	81±23	80±23	75±23	79±23	78±23	0.331[a]
**Stroke volume index (mL/m²)**	M	52±15	50±13	53±12	47±11†	45±14*†	<0.001[b]
	F	48±13	45±13	42±12*	45±13	44±13	0.003[a]
**Peripheral vascular resistance (dyn s/cm⁵)**	M	1140±456	1176±345	1058±311	1324±472*#†	1317±442†	<0.001[a]
	F	1286±370	1398±408*	1619±515*#	1630±649*#	1515±395*	<0.001[b]
**Peripheral vascular resistance index (dyn s m²/cm⁵)**	M	2208±889	2356±653	2121±576	2711±886*#†	2710±880*#†	<0.001[a]
	F	2179±607	2454±698*	2872±897*#	2893±1052*#	2692±591*	<0.001[b]
**Aortic PWV (m/s)**	M	6.60±1.24	7.19±1.30*	7.24±1.57*	7.94±1.56*#†	7.56±1.12*	<0.001[a]
	F	6.34±1.05	7.23±1.51*	8.61±1.65*#	8.19±1.90*#	7.49±1.29*†	<0.001[b]
**Adjusted aortic PWV (m/s)[1]**	M	7.20±1.17	7.35±1.24	7.15±1.54	7.26±1.52	7.27±1.07	0.790[a]
	F	6.90±1.05	7.15±1.41	7.82±1.69*#	6.83±1.78†	6.87±1.34†	<0.001[b]
**Aortic AIx (%)**	M	13.5±9.3	15.1±11.2	13.3±11.7	19.2±10.4*#†	14.8±12.4	<0.001[a]
	F	25.4±10.7	28.4±9.8*	30.9±9.1*	31.0±9.6*	29.9±7.9*	<0.001[a]
**Adjusted aortic AIx (%)[2]**	M	12.0±9.4	15.0±10.1	13.7±10.1	20.1±8.9*#†	16.2±12.0	<0.001[a]
	F	24.8±10.2	28.4±8.7*	31.9±7.6*#	31.9±7.9*#	30.8±6.9*	<0.001[b]
**Mean arterial pressure (mmHg)**	M	83±5	91±7*	96±7*#	108±10*#†	99±6*#§	<0.001[b]
	F	81±7	91±7*	102±8*#	111±10*#†	99±6*#§	<0.001[b]
**Heart rate (beats/min)**	M	61±11	63±10	65±12	67±11*#	68±12*	<0.001[a]
	F	64±9	66±10*	70±11*#	70±10*#	68±10	<0.001[a]

Data are means ± SD.

[a]One-way ANOVA, [b]Welch's ANOVA, [c]Kruskal-Wallis test.

*P<0.05 versus optimal BP, #P<0.05 versus prehypertension, †P<0.05 versus ISH, §P<0.05 versus SDH

[1]Data adjusted for mean arterial pressure. [2]Data adjusted for height and heart rate.

AIx=augmentation index; BP=blood pressure; F=female; IDH=isolated diastolic hypertension; ISH=isolated systolic hypertension; M=Male; SDH=systolic-diastolic hypertension; PWV=pulse wave velocity.

Table S5: Detailed haemodynamic variables across 5 blood pressure phenotypes for males and females >60 years

		Optimal BP	Prehypertension	ISH	SDH	IDH	P
**Cardiac output (L/min)**	M	4.9±1.7	5.4±1.6	5.4±1.3	5.7±1.7*	5.1±1.3	0.018[a]
	F	4.5±1.3	4.6±1.3	4.7±1.2	4.7±1.4	4.7±1.2	0.244[a]
**Cardiac index (L/min/m²)**	M	2.5±0.9	2.8±0.8	2.8±0.7	2.9±0.8	2.5±0.6	0.075[a]
	F	2.7±0.7	2.7±0.8	2.8±0.7	2.8±0.8	2.7±0.6	0.631[a]
**Stroke volume (mL)**	M	77±26	83±27	81±23	82±26	72±21	0.309[a]
	F	68±19	67±19	67±18	64±16	62±18	0.239[a]
**Stroke volume index (mL/m²)**	M	40±13	43±13	42±11	41±13	36±10	0.180[a]
	F	41±10	39±11	39±10	37±9	36±10	0.070[a]
**Peripheral vascular resistance (dyn s/cm⁵)**	M	1571±541	1494±483	1606±471#	1700±540#	1671±472	<0.001[c]
	F	1575±438	1755±530*	1820±501*	2052±656*#†	1810±466	<0.001[b]
**Peripheral vascular resistance index (dyn s m²/cm⁵)**	M	2995±1003	2875±903	3120±895#	3352±1022#	3319±783	<0.001[a]
	F	2627±706	2989±903*	3122±833*	3517±1157*#†	3130±746	<0.001[b]
**Aortic PWV (m/s)**	M	7.89±1.77	8.83±2.08*	10.08±2.47*#	10.16±2.54*#	8.52±1.17†§	<0.001[b]
	F	7.52±1.59	8.53±1.91*	9.78±2.22*#	10.37±2.39*#	8.35±1.43†§	<0.001[b]
**Adjusted aortic PWV (m/s)[1]**	M	8.90±1.81	9.27±2.04	9.85±2.46*#	9.14±2.35	8.42±1.07†	<0.001[b]
	F	8.50±1.52	8.77±1.94	9.35±2.21*#	9.13±2.37	7.96±1.35†§	<0.001[b]
**Aortic AIx (%)**	M	25.1±8.6	25.6±8.4	26.9±8.3	27.2±7.7	24.9±7.4	0.155[a]
	F	31.8±8.4	33.0±7.9	33.5±8.3	34.0±7.7	33.5±6.1	0.171[a]
**Adjusted aortic AIx (%)[2]**	M	24.2±7.5	25.2±7.1	26.7±7.1#	28.4±6.8*#	27.7±5.1	<0.001[c]
	F	31.3±7.5	32.7±6.8	33.8±6.5*	35.5±5.5*#	35.3±5.0	<0.001[c]
**Mean arterial pressure (mmHg)**	M	85±6	92±7*	101±8*#	111±10*#†	99±4*#§	<0.001[b]
	F	83±7	93±6*	101±8*#	112±9*#†	101±8*#§	<0.001[b]
**Heart rate (beats/min)**	M	62±7	64±10	65±10	68±11*#	72±12*	<0.001[b]
	F	65±10	66±9	68±10*	71±12*#	71±8	<0.001[a]

Data are means ± SD.

[a]One-way ANOVA, [b]Welch's ANOVA, [c]Kruskal-Wallis test.

*P<0.05 versus optimal BP, #P<0.05 versus prehypertension, †P<0.05 versus ISH, §P<0.05 versus SDH

[1]Data adjusted for mean arterial pressure. [2]Data adjusted for height and heart rate.

AIx=augmentation index; BP=blood pressure; F=female; IDH=isolated diastolic hypertension; ISH=isolated systolic hypertension; M=Male; SDH=systolic-diastolic hypertension; PWV=pulse wave velocity
